# Artificial Gauge Field and Topological Phase in a Conventional Two-dimensional Electron Gas with Antidot Lattices

**DOI:** 10.1038/srep15266

**Published:** 2015-10-16

**Authors:** Likun Shi, Wenkai Lou, F. Cheng, Y. L. Zou, Wen Yang, Kai Chang

**Affiliations:** 1 SKLSM, Institute of Semiconductors, Chinese Academy of Sciences, P.O. Box 912, Beijing 100083, China; 2 Beijing Computational Science Research Center, Beijing 100094, China

## Abstract

Based on the Born-Oppemheimer approximation, we divide the total electron Hamiltonian in a spin-orbit coupled system into the slow orbital motion and the fast interband transition processes. We find that the fast motion induces a gauge field on the slow orbital motion, perpendicular to the electron momentum, inducing a topological phase. From this general designing principle, we present a theory for generating artificial gauge field and topological phase in a conventional two-dimensional electron gas embedded in parabolically graded GaAs/In_*x*_Ga_1−*x*_As/GaAs quantum wells with antidot lattices. By tuning the etching depth and period of the antidot lattices, the band folding caused by the antidot potential leads to the formation of minibands and band inversions between neighboring subbands. The intersubband spin-orbit interaction opens considerably large nontrivial minigaps and leads to many pairs of helical edge states in these gaps.

Exploring of various topological quantum states is always one of the central issue of condensed matter physics^1^,^2^,^3^. Topological insulators (TIs)^4^, a new class of solids, posses unique properties such as robust gapless helical edge or surface states and exotic topological excitations^4^,^5^,^6^,^7^,^8^,^9^,^10^,^11^,^12^,^13^,^14^,^15^,^16^,^17^,^18^,^19^,^20^,^21^,^22^,^23^,^24^,^25^,^26^. The helical edge states of two-dimensional (2D) TIs are protected strictly against elastic backscattering from nonmagnetic impurities. This feature leads to dissipationless conducting channels and therefore is promising for possible applications in spintronics, quantum information, thermoelectric transport and on-chip interconnection in integrated circuit. These novel applications require large nontrivial gaps, which suppress the coupling between the edge and bulk states, leading to dissipationless edge transport. For this purpose, there is an ongoing search for feasible realizations of various narrow gap materials containing heavy elements, e.g., CdTe/HgTe/CdTe quantum wells (QWs)^7^,^8^,^9^, and Tin film^22^. However, fabrication of high-quality samples of these proposed structures still remains a challenging task, requiring precise control for material growth.

In this work, we demonstrate that conventional semiconductor GaAs/In_*x*_Ga_1−*x*_As/GaAs two-dimensional electron gas (2DEG) with antidot lattices can be driven into the TI phase. The 2DEGs provide a promising playground for realizing TI states with quite large nontrivial gap (~20 meV) operating at liquid nitrigen temperature regime, instead of searching new materials containing heavy atoms. We first present a general analysis for generating an artificial gauge field in a semiconductor 2DEG, then we demonstrate band inversion between *neighboring subbands* because of *inter-subbands* spin-orbit interaction (ISOI) utilizing antidot lattices created by well-developed semiconductor etching technique, and generate the TI phase with many pairs of helical edge states. This suggests a completely new method to generate topological phase in conventional semiconductor 2DEGs without strong spin-orbit interaction (SOI), at liquid nitrigen temperature regime.

## Results

### General design principle: gauge field from Born-Oppenheimer approximation

First we discuss the emergence of an artificial gauge field in a system of electrons in a 2D system described by a low-energy single-particle Hamiltonian 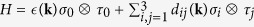, where 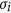 and 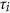 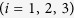 are Pauli matrices describing the electron spin and the conduction 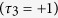 and valence 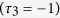 bands, respectively, and 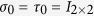 are identity matrices. Taking 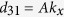, 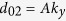, 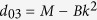 and other 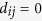, we obtain the Bernevig-Hughes-Zhang (BHZ) Hamiltonian for 2D TIs^7^. Neglecting the band index *τ*, and taking 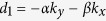, 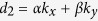, *d*_3_ = 0, we get the Hamiltonian for a 2DEG with Rashba and Dresselhaus SOIs, where *α* and *β* are the strengths of Rashba and Dresselhaus SOIs, respectively. Next, we divide the total Hamiltonian at the band edge into the intra-band (typical energy scale is about 10^−2^ meV), slow part 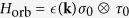 and the inter-band (typical energy scale is about 1 ~ 10^2^ meV), fast part 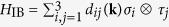, which usually arises from the SOIs or ISOIs in real materials. The eigenstate of the total Hamiltonian can be decomposed into the fast and slow components: 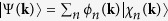, where 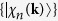 are eigenstates of the fast part *H*_IB_ and 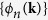 describe the slow part. The fast spin dynamics compared with the slow orbital motion allow us to make the Born-Oppenhenmer approximation, i.e., neglecting the coupling between different 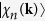, and derive an effective Hamiltonian governing the slow orbital motion 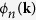



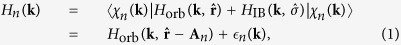


where 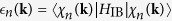 acts as an effective potential that seperates different bands 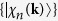 and 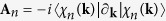 is a gauge potential in the momentum space of the slow orbital motion, due to the interband coupling to the fast spin dynamics^27^,^28^. For the BHZ Hamiltonian, the gauge potential **A**_*n*_ leads to an effective Lorentz force 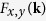 in the momentum space perpendicular to the electric field **E**:


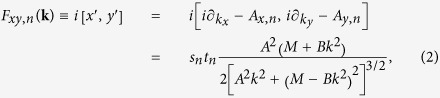


where *s*_*n*_ = ±1 denotes spin up or down state while *t*_*n*_ = ±1 denotes the conduction or valence band, respectively (*n* = 1, 2, 3, 4). The Chern number 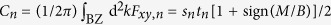 is obtained by integrating the field strength *F*_*xy,n*_ in the Brillouin zone. The sign change in *M* would induce a change of the Chern number by 1, which corresponds to the topological phase transition^29^.

**Figure 1:**
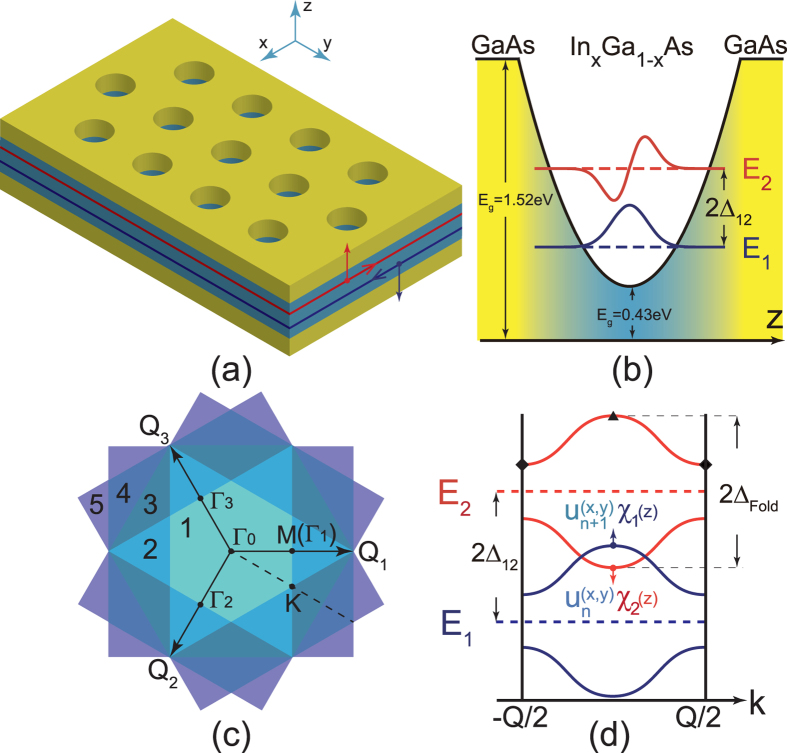
Schematic of the proposed structure and its energy bands. (**a**) A GaAs/In_*x*_Ga_1−*x*_As/GaAs parabolically graded QW with an antidot lattice, which can be created by etching technique. (**b**) Band profile and the first and second subbands of the parabolically graded QW. (**c**) Brillouin zone folding induced by a triangular antidot lattice. The numbers 1–5 denote the first to the fifth Brillouin zones of the antidot lattice. (**d**) Minibands of the antidot lattice from folding the first and second subbands of the QW (*Q* = 2*π*/*a* and *a* is the antidot lattice constant). Note that band inversion occurs between neighboring minibands.

For a 2DEG with Rashba and Dresselhaus SOIs, we find 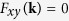, which means that the Chern number vanishes in 2DEG with SOIs. Comparing the Hamiltonian of 2D TIs to that of 2DEGs with SOIs, one can see clearly that 2D TIs posses an additional degree of freedom: the band index *τ*. In order to generate the gauge field and realize TI phases in a 2DEG, one needs to create minibands and band inversion in 2DEGs. Based on the above designing principle, we will create topological phase in conventional semiconductor 2DEG. This is the first demonstration of the formation of a TI phase in the *s-like* band systems, i.e., a 2DEG with nanostructured antidot lattice shown schematically in Fig. 1(a).

### Topological phase transition in two-dimensional electron gas: effective model

Nanostructured antidot lattices, consisting of periodically arranged holes that are etched in a 2DEG, form a strongly repulsive egg-carton-like periodic potential in a 2DEG^30^,^31^,^32^,^33^,^34^,^35^,^36^,^37^. This artificial crystals lead to a wide variety of phenomena, for instance, Weiss oscillation, chaotic dynamics of electrons, the formation of an electronic miniband structure and massless Dirac fermions. At low temperatures, the mean free path of electrons is much longer than the period of antidot lattices ranging from 10 to 100 nanometers. The modulated periodic potential can also be created by electron beam lithography electrodeposition and periodic arrays of metallic nanodots can be realized on semiconductor surfaces. Due to elastic strains producing these dots, a sufficiently strong piezoelectric potential modulation results in miniband effects in the underlying 2DEG^32^,^33^. Very recently, a honeycomb lattice of coronene molecules was created by using a cryogenic scanning tunneling microscope on a Cu(111) surface to construct artificial graphene-like lattice with the lattice constant approaching 5 nm^37^.

We consider the 2DEG in a GaAs/In_*x*_Ga_1−*x*_As/GaAs parabolically graded QW, which was fabricated successfully before^38^,^39^,^40^, with a triangular antidot lattice (see Fig. 1(a). Before going to the numerical calculation, we first give a clear physical picture for the emergence of a TI phase in this 2DEG system upon nanostructuring with antidot lattice. The simplest description of the 2DEG system is obtained by reducing the eight-band Kane model to the lowest conduction subbands of the QW (see Methods). This gives the Hamiltonian for the 2DEG with periodic antidot lattice potential *V*(*x*, *y*):


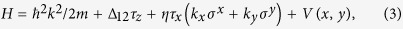


where 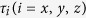 are Pauli matrices describing the first and second QW subbands 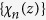 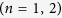 of effective mass *m*, and 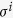 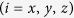 refer to the electron spin. The second term 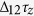 comes from the energy difference 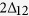 between the first and second subbands [see Fig. 1(b)]. The third term 
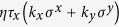
 describes the inter-subbands SOI (ISOI) obtained from the eight-band Kane model using the Löwdin perturbation theory^41^ (see Methods). The coupling strength *η* is


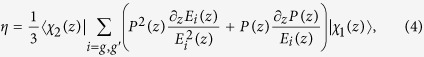


where 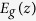 and 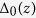 are the band gap and spin split-off splitting in the QW region, 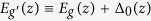, and 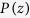 is the Kane matrix element. The SOIs in 2DEGs usually come from the asymmetry of the QWs, i.e., Rashba SOI. Surprisingly, the ISOI can appear in a symmetric parabolically graded QW, behaving like a hidden SOI. From Eq. (4), one can see that the ISOI arises from the spatial variations of the bandgap 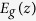, the Kane matrix 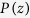, and the intrinsic SOI 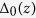, i.e., the variation of the concentration of *In* component, which behaves like an effective local electric field. This local electric field would not push the electron and the hole states to the left and right sides of the QW, but it can induce a considerably large ISOI hidden in symmetric QWs. The initial 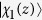 and final states 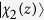 are neighboring subbands having opposite parity, while the variations of 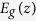, 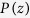 and 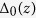 in a symmetric QW are odd. This means that the ISOI can exist in symmetric QWs. Here we neglect Dresselhaus SOI term which is proportional to 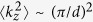 (d is the thickness of the QW) because in our proposal the QW thickness is quite large (300A), therefore the strength of Dresselhaus SOI is quite weak. We would also emphasize that the Dresselhaus SOI only exists in the same subband, which is an intra-subband interaction and will not affect our above analysis and topological nontrivial gap in such system, i.e., the band inversion between two adjacent subbands.

The first and second subbands both form minibands due to the Brillouin zone folding caused by the antidot lattice. The band inversion could occur between the two adjacent minibands of the first subband 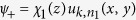 and the second subband 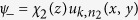 (see Fig. 1(d), where 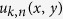 is the *n* th miniband formed by the antidot lattice. We model the triangular antidot lattice potential 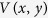 by a periodic potential 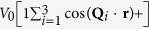 with potential height *V*_0_^32^,^41^,^42^,^43^,^44^, 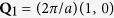, 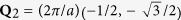, 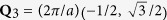 and *a* is the triangular antidot lattice constant (see Methods).

To describe the four minibands (two spin-degenerate minibands) 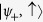, 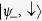, 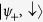, 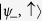 involved in the band inversion, we treat other electron and hole minibands by Löwdin perturbation theory and reduce the eight-band Kane **k** · **p** model to the following effective Hamiltonian within the basis 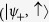, 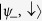, 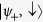, 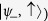:


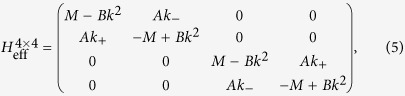


(see Methods and Supplementary Note 2), which assumes the same form as the *D* = 0 BHZ Hamiltonian. Here 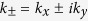, 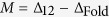 [see Fig. 1(b)], 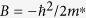, and *A* characterize the ISOI strength between neighboring minibands with opposite spin. This Hamiltonian obviously has a *Z*_2_ topological phase when *M* < 0, corresponding to band inversion.

### Topological phase transition in two-dimensional electron gas: numerical calculation

**Figure 2:**
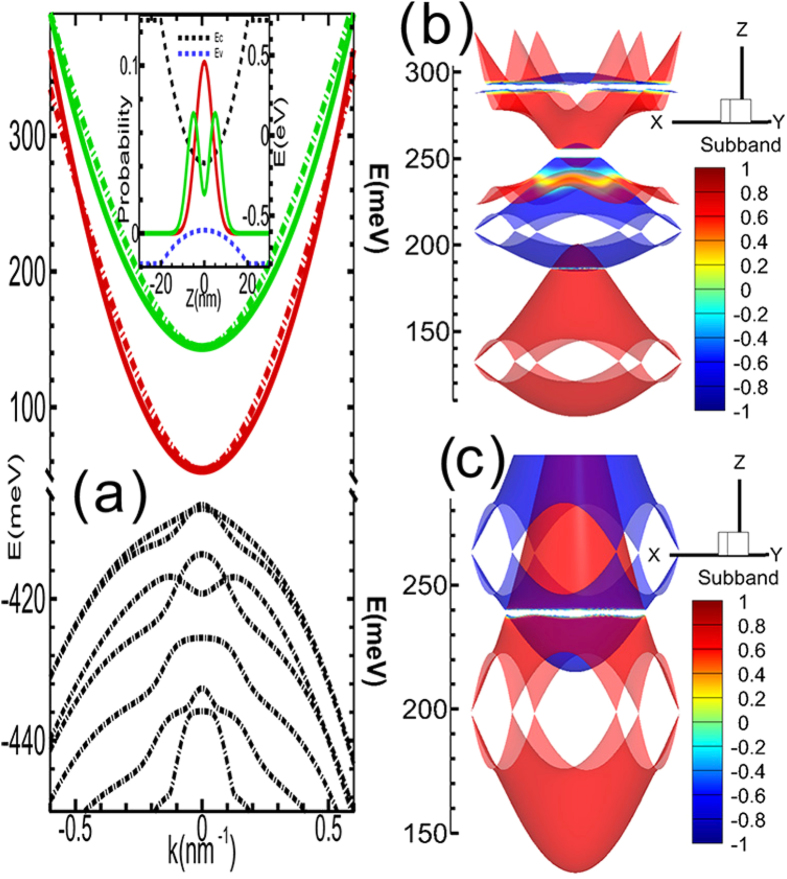
Subbands of the QW and minibands of the antidot lattice. (**a**) Band structure of a GaAs/In_*x*_Ga_1−*x*_As/GaAs parabolically graded QW from eight-band Kane model. The red and blue dashed (solid) curves denote the first and second subbands from the eight-band Kane model (the reduced four-band model). The inset shows the spatial distributions of the first and second subbands. (**b**,**c**): minibands of a parabolically graded QW for two different triangular antidot lattices: the lattice constants and barrier heights *V*_0_ of the antidot lattices are *a* = 17 nm, *V*_0_ = 200 meV for (**b**) and 12.5 nm, 300 meV for (**c**). The minibands are inverted and the minigaps are opened.

We employ the eight-band Kane **k** · **p** model to calculate the subband structure with SOIs in a 40-nm-thick GaAs/In_*x*_Ga_1−*x*_As/GaAs parabolically graded QWs^38^,^39^, as plotted in Fig. 2(a). The energy difference between the minima of the first and second subbands at Γ point is about 90 meV (see Fig. 2(a). In order to calculate the miniband structures caused by an in-plane periodic potential induced by the triangular antidot lattice, we reduce the eight-band model to an effective four-band **k** · **p** Hamiltonian by including the lowest 20 electron subbands and 54 highest hole subbands in the QW, to reproduce the energy dispersions of the first and second subbands calculated from the eight-band Kane model (see Fig. 2a). The parameters in the four-band Hamiltonian is given Supplementary Note 2. The minibands from the four-band **k** · **p** Hamiltonian are shown in Fig. 2(b,c). These minibands originates from folding the first and second subbands of the QW into the first Brillouin zone of the antidot lattice [Fig. 1(c)]. By tuning the antidot lattice constant *a* and the potential height *V*_0_, i.e., the etching depth of the antidot lattice, many band inversions appear between these minibands, which can be clearly seen in Fig. 2(b,c). The minigaps between these minibands are opened by the ISOI shown in Eq. (4) [see Fig. 2(b,c)].

To demonstrate that these minigaps are topologically nontrivial, we determine the parity of each miniband at the four time-reversal invariant momenta^11^ Γ_i_ (*i* = 0, 1, 2, 3) in the first Brillouin zone shown in Fig. 1(c). For the lowest *N* spin-degenerate minibands being occupied, the *Z*_2_ invariant is given by 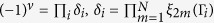, where 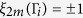 is the parity of the 2*m*th occupied miniband at Γ_i_. Our calculation gives 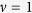 at all the minigaps, which proves the whole system is in the quantum spin-Hall phase (see Methods).

**Figure 3:**
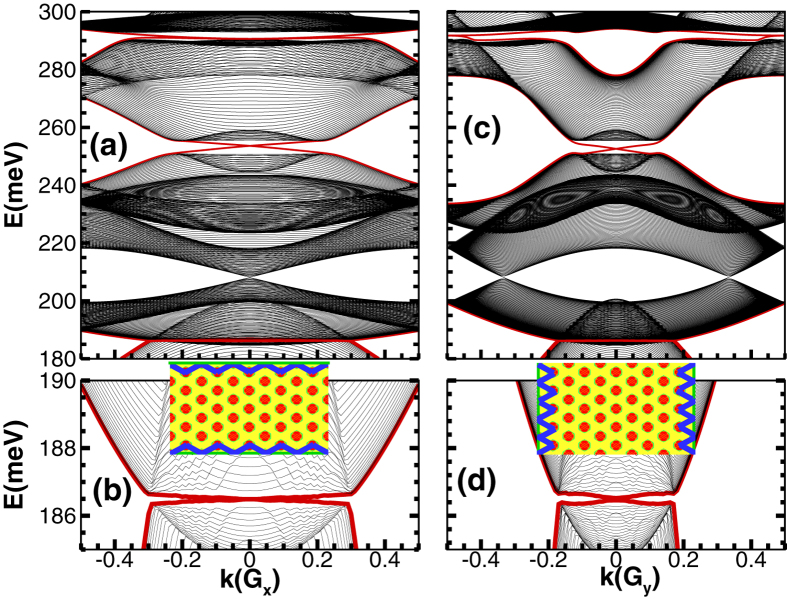
Edge states in nontrivial minigaps of a Hall bar structure of the antidot lattice. The Hall bar orientation is along the *x* axis (**a**) or *y* axis (**c**), as sketched in the insets of (**b**,**d**), respectively. The lower panels (**b**,**d**) amplify the lowest nontrivial minigaps and gapless topological edge states. The lattice constant of the antidot lattice is *a* = 17 nm. The blue curves indicate the spatial distributions of the edge states.

Next, we demonstrate the emergence of topological edge states upon etching the QW into a Hall bar structure along two different directions (*x* axis and *y* axis). As shown in Fig. 3, a pair of topological helical edge states appear inside each nontrivial minigap. For example, we can see topological helical edge states in the lowest two nontrivial minigaps near ~186.5 meV and ~255 meV, respectively. The helical edge state pairs in these minigaps would lead to higher conductance plateaus as the Fermi energy increases by increasing the doping level. The helical edge states do not overlap with the bulk states, making it possible to be detected experimentally.

**Figure 4:**
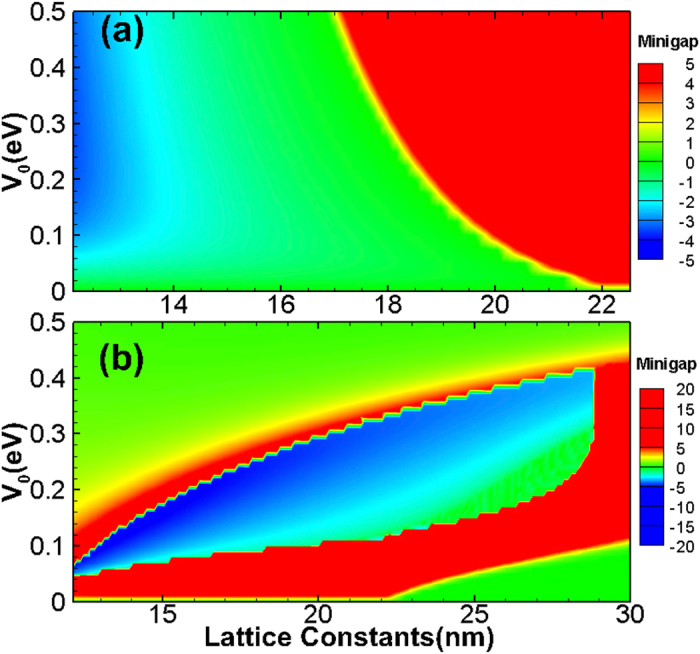
Phase diagrams of the antidot lattice on a GaAs/In_*x*_Ga_1−*x*_As/GaAs parabolically graded QWs. The lowest (**a**) and second (**b**) minigap vs. antidot lattice constant *a* and potential height *V*_0_. Negative (Positive) minigap indicates TI phase (normal phase).

The lowest nontrivial minigaps is quite small (about 0.5 meV), but the second minigap is larger (about 5 meV). By tuning the period and potential height of the antidot lattice, the nontrivial minigaps can be significantly enhanced [see Fig. 4(a,b)]. For example, the lowest minigaps can be enhanced to 5 meV, which is already comparable with that in HgTe and InAs/GaSb QW systems (~10 meV)^8^,^9^. The second minigaps can approach 20 meV, which means the TI phase can be realized at liquid nitrigen temperature regime. From Fig. 4(a,b), one can see that the lowest nontrivial minigap is closed as the lattice constant *a* increases, but the second higher nontrivial minigap survives, i.e., the TI phase can exist even at large lattice constants, e.g., 25 nm. We remark that the randomness of the size and position, i.e., disorder effect, might smear our the nontrivial minigap. However, the previous works^45^,^46^,^47^ demonstrated that the disorder effect would not cancel topological phase, instead, it will lead to topological Anderson insulator phase where the edge states can exist even for very strong disorder strength, which is much larger than the bandgap.

### Experimental detection scheme

One way to detect the aforementioned edge states (shown in Fig. 3) is the standard four terminal measurements as demonstrated in previous works^8^,^9^. In contrast to HgTe and InAs/GaSb quantum well systems, there are many pairs of helical edge states in our system between these inverted minibands, which leads to higher plateaus with increasing the Fermi energy. Another possible way is microwave impedance microscopy which makes spatial-resolved nano-scale images (<100 nm) of the conductivity and permittivity of a sample^48^. The unoccupied edge states in higher minigaps can be detected using the angle-resolved photonemission technique^49^, which has already been successfully applied to identify occupied and unoccupied surface states in Bi_2_Se_3_ and Bi_2_Te_*x*_Se_3_^49^,^50^,^51^.

## Discussion

Our proposal is based on a general analysis about the electron orbital motion in TIs. By using the Born-Oppenheimer approximation, we find that the fast motion will induce a spin-dependent gauge field on slow orbital motion. Based on this general analysis, we demonstrate theoretically the TI phase in a conventional 2DEG embedded in a symmetric parabolically graded GaAs/In_*x*_Ga_1−*x*_As/GaAs QW, with antidot lattices created by well-developed etching technique. The key point is to create a ISOI in a symmetric quantum well, in contrast to conventional SOI in asymmetric QWs. This hidden ISOI in symmetric QWs induces a spin-dependent effective Lorentz force on the electrons, and generates the TI phases in such system. Interestingly, such ISOI exists in conventional semiconductors with a positive bandgap, i.e., normal band structures can generate quite large nontrivial gaps approaching 20 meV. This make it possible to observe the quantum spin Hall effect in liquid nitrigen temperature regime.

So far, all members of TI family are narrow bandgap systems containing heavy atoms. Our proposal breaks this constraint, and makes it possible to realize TI phase in conventional semiconductor 2DEG using the well-developed semiconductor fabrication techniques. The presence of the TI phase in parabolically graded QWs with antidot lattice can largely advance the application of this new quantum state in existing electronics and optoelectronics devices. The general designing principle proposed in this work, i.e., the gauge field acting on slow orbital motion induced by interband coupling, paves a new way for generating nontrivial topological phases, such as quantum spin Hall phase and even quantum anomalous Hall phases by doping magnetic ions, in conventional semiconductor 2DEGs, and suggests a promising approach to integrate it in well developed semiconductor electronic devices.

## Methods

### Effective spin-orbit coupling in a quantum well

For a symmetric quantum well grown along (001) direction (the *z* axis), effective spin-orbit coupling exists between subbands with opposite parities. This effective spin-orbit coupling comes from interband coupling and can be understand by reducing the 8 × 8 Kane Hamiltonian to a 2 × 2 effective Hamiltonian.

To the first order of *k*, the 8 × 8 Kane Hamiltonian in the basis 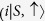, 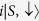,  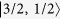,  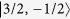, 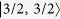, 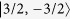, 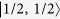, 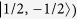 around the Γ point is


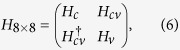


where 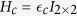 and 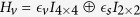 are 2 × 2 and 6 × 6 diagonal part for conduction and valence bands, and the 2 × 6 matrix


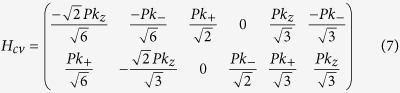


represents the interband coupling. Specifically, 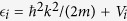 is the kinetic energy plus the total potential for the conduction/valence/spin-split (*i* = *c*/*v*/*s*) bands, with 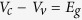 the band gap and 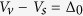 the band off set. 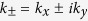 and 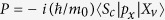 parameterize the interband coupling.

The eigenvalue problem can be expressed as


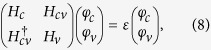


where 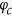 is a two-component spinor for conduction bands and 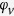 is a six-component spinor for valence bands. Since we focus on the conduction bands, 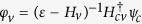 can be eliminated and gives the effective Schrödinger-type equation 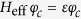, with 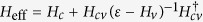 for conduction bands. Without loss of generality, we assume the quantum well is non-uniform only along the *z* direction, e.g., a parabolically graded QW. By straightforward algebra, we have 
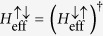
 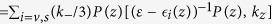, where 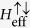 and 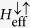 represent the effective spin-orbit coupling between the spin up and down electron.

Since we focus on the lowest conduction subbands, we have 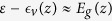 and 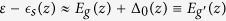. Because 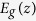 and 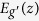 are much larger than the subband energies in the wide QWs under consideration, we keep the zero-th order terms 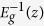 and 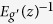 in the expansion, and project the spin-orbit coupling operator 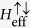 into the two lowest spin-degenerate subbands 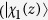, 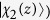 to obtain the ISOI 
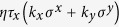
, where *η* is given in Eq. (4), *σ*^*i*^ denotes the real electron spin, and *τ*_*i*_ refers to the Pauli matrix describing the the subband index.

### Band edge wave functions in folded Brillouin zone

We consider a parabolically graded QW in the presence of an antidot lattice, which can be generally described by a potential 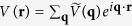 with the lattice periodicity.

For a triangular antidot lattice, the reciprocal lattice vectors in the hexagonal Brillouin zone are 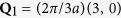, 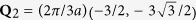, 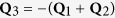. The envelope functions of the lowest miniband at the band edge (*k* = 0, Γ point) is 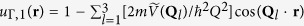. For higher minibands, their envelope functions 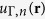 (*n* = 2, 3, 4, 5, 6, 7) at the band edge are linear combinations of the six wave vector components 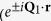, 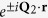, 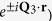, e.g., 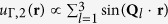 and 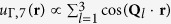. The most important minibands are 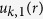, 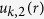 and 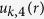: the lowest nontrivial minigap occurs between 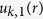 and 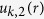, and the second nontrivial minigap occurs between 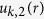 and 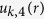.

### Effective BHZ Hamiltonian near Γ point

The lowest two subbands 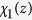 and 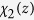 in a parabolically graded QW have even and odd opposite parities, an effective spin-orbit interaction 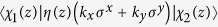 appears. When the Brillouin zone is folded by the triangular anti-dot lattice, the lowest nontrivial minigap appears between the miniband pair 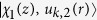 and 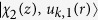, i.e., the second miniband of the first subband and the first miniband of the second subband. The second nontrivial minigap appears between the miniband pair 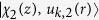 and 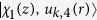, i.e., the second miniband of the second subband and the fourth miniband of the first subband. To obtain an effective Hamiltonian near each minigap, we project the Hamiltonian 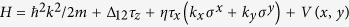 onto the corresponding miniband pair and obtain an effective BHZ model Eq. (5) in the basis 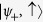, 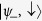, 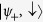, 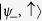, where 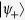 is the miniband above 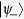 by 2*M* at the Γ point, 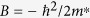 characters the band dispersions with the effective mass m* near the band edge, and *A* characterize the intersubband spin-orbit coupling. At the Γ point


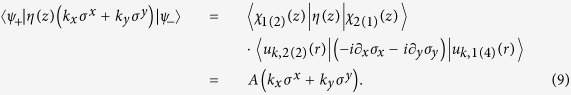


The accurate coupling strength can be estimated by numerical calculating based on the eight-band Kane model.

For BHZ model, a *Z*_2_ topological transition from the normal phase to the topological insulator phase would occur when 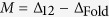 [see Fig. 1(b)] changes sign from positive to negative, which can be controlled by adjusting the lattice constants and etching depths of antidots.

### Verification of non-trivial *Z*_2_ topological invariant

Topological insulators with dissipationless edge states and ordinary insulators are distinguished by different *Z*_2_ invariants. For 2D systems, Fu and Kane^11^ have shown that the *Z*_2_ invariant can be determined from the parity of the occupied band at the four time-reversal invariant momenta in the Brillouin zone. The *Z*_2_ invariant 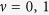, which distinguishes the quantum spin-Hall phase in two dimensions, is given by


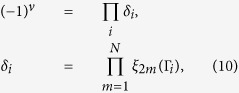


where 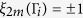 is the parity eigenvalue of the 2*m*th occupied energy band at the time-reversal invariant point Γ_*i*_, which shares the same eigenvalue 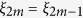 with its Kramer degenerate partner. The four time-reversal invariant points 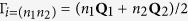, where 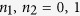. The calculated parity eigenvalue of the 2*m*th (*m* = 1, 2, 3, 4, 5, 6) occupied energy band at Γ_ι_ are listed:


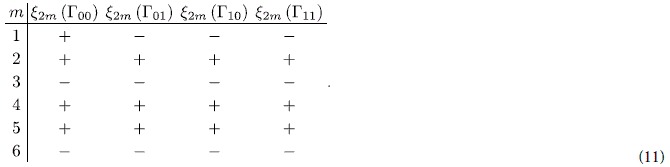


From the above calculation, we can confirm that the *Z*_2_ invariant *ν* = 1 at the 2*m*th (*m* = 2, 5, 6) occupied band where the minigaps open, and the system enters the TI phase and the dissipationless edge states appear.

## Additional Information

**How to cite this article**: Shi, L. *et al*. Artificial Gauge Field and Topological Phase in a Conventional Two-dimensional Electron Gas with Antidot Lattices. *Sci. Rep*. **5**, 15266; doi: 10.1038/srep15266 (2015).

## Supplementary Material

Supplementary Information

## References

[b1] Thouless D. J., Kohmoto M., Nightingale M. P. & Den Nijs M. Quantized Hall Conductance in a Two-Dimensional Periodic Potential. Phys. Rev. Lett. 49, 405 (1982).

[b2] Niu Q., Thouless D. J. & Wu Y. S. Quantized Hall conductance as a topological invariant. Phys. Rev. B 31 3372 (1985). 10.1103/physrevb.31.3372 9936224

[b3] Haldane F. D. M. Model for a Quantum Hall Effect without Landau Levels: Condensed-Matter Realization of the “Parity Anomaly”. Phys. Rev. Lett. 61, 2015 (1988). 10038961 10.1103/PhysRevLett.61.2015

[b4] Hasan M. Z. & Kane C. L. Colloquium: Topological insulators. Rev. Mod. Phys. 82, 3045 (2010).

[b5] Qi X. L. & Zhang S. C. Topological insulators and superconductors. Rev. Mod. Phys. 83, 1057 (2011).

[b6] Kane C. L. & Mele E. J. Z_2_ Topological Order and the Quantum Spin Hall Effect. Phys. Rev. Lett. 95, 146802 (2005). 16241681 10.1103/PhysRevLett.95.146802

[b7] Bernevig B. A., Hughes T. L. & Zhang S. C. Quantum Spin Hall Effect and Topological Phase Transition in HgTe Quantum Wells. Science 314, 1757 (2006). 17170299 10.1126/science.1133734

[b8] König M. . Quantum Spin Hall Insulator State in HgTe Quantum Wells. Science 318, 766 (2007). 17885096 10.1126/science.1148047

[b9] Knez I., Du R. R. & Sullivan G. Evidence for Helical Edge Modes in Inverted InAs/GaSb Quantum Wells. Phys. Rev. Lett. 107, 136603 (2011). 22026882 10.1103/PhysRevLett.107.136603

[b10] Fu L., Kane C. L. & Mele E. J. Topological Insulators in Three Dimensions. Phys. Rev. Lett. 98, 106803 (2007). 17358555 10.1103/PhysRevLett.98.106803

[b11] Fu L. & Kane C. L. Topological insulators with inversion symmetry. Phys. Rev. B 76, 045302 (2007).

[b12] Hsieh D. . A topological Dirac insulator in a quantum spin Hall phase. Nature 452, 970 (2008). 18432240 10.1038/nature06843

[b13] Chen Y. L. . Experimental Realization of a Three-Dimensional Topological Insulator, Bi_2_Te_3_. Science 325, 178 (2009). 19520912 10.1126/science.1173034

[b14] Xia Y. . Observation of a large-gap topological-insulator class with a single Dirac cone on the surface. Nature Phys. 5, 398 (2009).

[b15] Lin H. . Half-Heusler ternary compounds as new multifunctional experimental platforms for topological quantum phenomena. Nature Mater. 9, 546 (2010). 20512153 10.1038/nmat2771

[b16] Franz M. Topological insulators: Starting a new family. Nature Mater. 9, 536 (2010). 20512152 10.1038/nmat2783

[b17] Yang K., Setyawan W., Wang S., Nardelli M. B. & Curtarolo S. A search model for topological insulators with high-throughput robustness descriptors. Nature Mater. 11, 614 (2012). 22581314 10.1038/nmat3332

[b18] Chadov S. . Tunable multifunctional topological insulators in ternary Heusler compounds, Nature Mater. 9, 541 (2010). 20512154 10.1038/nmat2770

[b19] Xiao D. . Half-Heusler Compounds as a New Class of Three-Dimensional Topological Insulators. Phys. Rev. Lett. 105, 096404 (2010). 20868181 10.1103/PhysRevLett.105.096404

[b20] Sushkov O. P. & Castro Neto A. H. Topological Insulating States in Laterally Patterned Ordinary Semiconductors. Phys. Rev. Lett. 110, 186601 (2013). 23683229 10.1103/PhysRevLett.110.186601

[b21] Xiao D., Zhu W. G., Ran Y., Nagaosa N. & Okamoto S. Interface engineering of quantum Hall effects in digital transition metal oxide heterostructures. Nature Commun. 2, 596 (2011). 22186892 10.1038/ncomms1602

[b22] Xu Y. . Large-Gap Quantum Spin Hall Insulators in Tin Films. Phys. Rev. Lett. 111, 136804 (2013). 24116803 10.1103/PhysRevLett.111.136804

[b23] Li J. & Chang K. Electric field driven quantum phase transition between band insulator and topological insulator. Appl. Phys. Lett. 95, 222110 (2009).

[b24] Miao M. S. . Polarization-Driven Topological Insulator Transition in a GaN/InN/GaN Quantum Well. Phys. Rev. Lett. 109, 186803 (2012). 23215311 10.1103/PhysRevLett.109.186803

[b25] Zhang D., Lou W. K., Miao M. S., Zhang S. C. & Chang K. Interface-Induced Topological Insulator Transition in GaAs/Ge/GaAs Quantum Wells. Phys. Rev. Lett. 111, 156402 (2013). 24160616 10.1103/PhysRevLett.111.156402

[b26] Hu J., Alicea J., Wu R. Q. & Franz M. Giant Topological Insulator Gap in Graphene with 5*d* Adatoms. Phys. Rev. Lett. 109, 266801 (2012). 23368597 10.1103/PhysRevLett.109.266801

[b27] Wilczek F. & Zee A. Appearance of Gauge Structure in Simple Dynamical Systems, Phys. Rev. Lett. 52, 2111 (1984).

[b28] Sun C. P. & Ge M. L. Generalizing Born-Oppenheimer approximations and observable effects of an induced gauge field. Phys. Rev. D 41, 1349 (1990). 10.1103/physrevd.41.1349 10012480

[b29] Lindner N. H., Refael G. & Galitski V. Floquet topological insulator in semiconductor quantum wells. Nature Phys. 7, 490 (2011).

[b30] Weiss D. . Electron pinball and commensurate orbits in a periodic array of scatterers. Phys. Rev. Lett. 66, 2790 (1991). 10043617 10.1103/PhysRevLett.66.2790

[b31] Eroms J. . Skipping orbits and enhanced resistivity in large-diameter InAs/GaSb antidot lattices. Phys. Rev. B 59, 7829(R) (1999).

[b32] Albrecht C. . Fermiology of Two-Dimensional Lateral Superlattices. Phys. Rev. Lett. 83, 2234 (1999).

[b33] Albrecht C. . Evidence of Hofstadter’s Fractal Energy Spectrum in the Quantized Hall Conductance. Phys. Rev. Lett. 86, 147 (2001). 11136115 10.1103/PhysRevLett.86.147

[b34] Bittkau K., Menk Ch., Heyn Ch., Heitmann D. & Hu C. M. Far-infrared photoconductivity of electrons in an array of nanostructured antidots. Phys. Rev. B 68, 195303 (2003).

[b35] Yuan Z. Q., Yang C. L., Du R. R., Pfeiffer L. N. & West K. W. Microwave photoresistance of a high-mobility two-dimensional electron gas in a triangular antidot lattice. Phys. Rev. B 74, 075313 (2006).

[b36] Park C. H. & Louie S. G. Making Massless Dirac Fermions from a Patterned Two-dimensional Electron Gas. Nano Lett. 9, 1793 (2009). 19338276 10.1021/nl803706c

[b37] Wang S., Tan L. Z., Wang W., Louie S. G. & Lin N. Manipulation and Characterization of Aperiodical Graphene Structures Created in a Two-Dimensional Electron Gas. Phys. Rev. Lett. 113, 196803 (2014). 25415917 10.1103/PhysRevLett.113.196803

[b38] Sacedón A. . Design of InGaAs linear graded buffer structures. Appl. Phys. Lett. 66, 3334 (1995).

[b39] Liang J., Chua Y. C., Manasreh M. O., Marega E. Jr. & Salamo G. J. Broad-band photoresponse from InAs quantum dots embedded into InGaAs graded well, IEEE Electron Device Letters 26, 631 (2005).

[b40] Dai N. . Band offset determination in the strained-layer InSb/Al_*x*_In_1−*x*_Sb system. Appl. Phys. Lett. 76, 3905 (2000).

[b41] Winkler R. Spin-Orbit Coupling Effects in Two-Dimensional Electron and Hole Systems. Springer, Berlin (2003).

[b42] Lorke A., Kotthaus J. P. & Ploog K. Magnetotransport in two-dimensional lateral superlattices. Phys. Rev. B 44, 3447(R) (1991). 10.1103/physrevb.44.3447 9999965

[b43] Weiss D. . Quantized periodic orbits in large antidot arrays. Phys. Rev. Lett. 70, 4118 (1993). 10054051 10.1103/PhysRevLett.70.4118

[b44] Neudert R., Rotter P., Rössler U. & Suhrke M. Magnetotransport in rectangular antidot superlattices. Phys. Rev. B 55, 2242 (1997).

[b45] Jian L., Rui-Lin C., Jain J. K. & Shun-Qing S. Topological Anderson Insulator. Phys. Rev. Lett. 102, 136806 (2009). 19392389 10.1103/PhysRevLett.102.136806

[b46] Groth C. W., Wimmer M., Akhmerov A. R., Tworzydlo J. & Beenakker C. W. J. Theory of the Topological Anderson Insulator. Phys. Rev. Lett. 103, 196805 (2009). 20365944 10.1103/PhysRevLett.103.196805

[b47] Altland A., Bagrets D., Fritz L., Kamenev A. & Schmiedt H. Quantum Criticality of Quasi-One-Dimensional Topological Anderson Insulators. Phys. Rev. Lett. 112, 206602 (2014).

[b48] Lai K. . Imaging of Coulomb-Driven Quantum Hall Edge States. Phys. Rev. Lett. 107, 176809 (2011). 22107561 10.1103/PhysRevLett.107.176809

[b49] Sobota J. A. . Direct Optical Coupling to an Unoccupied Dirac Surface State in the Topological Insulator Bi_2_Se_3_. Phys. Rev. Lett. 111, 136802 (2013). 24116801 10.1103/PhysRevLett.111.136802

[b50] Ueda Y. . Photoemission and inverse-photoemission studies of Bi_2_Y_3_ (Y = S, Se, Te)semiconductors. J. Electron Spectrosc. Relat. Phenom. 101, 677 (1999).

[b51] Niesner D. . Unoccupied topological states on bismuth chalcogenides. Phys. Rev. B 86, 205403 (2012).

