# Corrigendum: Artificial Gauge Field and Topological Phase in a Conventional Two-dimensional Electron Gas with Antidot Lattices

**DOI:** 10.1038/srep28215

**Published:** 2016-07-07

**Authors:** Likun Shi, Wenkai Lou, F. Cheng, Y. L. Zou, Wen Yang, Kai Chang

Scientific Reports 5: Article number: 1526610.1038/srep15266; published online: 10162015; updated: 07072016

The Authors neglected to cite previous studies related to the existence of intersubband spin-orbit coupling and the use of intersubband spin-orbit coupling to create topological insulator in double quantum wells with antidote lattices. The additional references are listed below as references 1 and 2, and should appear in the text as below.

In the Introduction section,

“In this work, we demonstrate that conventional semiconductor GaAs/In_*x*_Ga_1−*x*_As/GaAs two-dimensional electron gas (2DEG) with antidot lattices can be driven into the TI phase.”

should read:

“In this work, we demonstrate that conventional semiconductor GaAs/In_*x*_Ga_1−*x*_As/GaAs two-dimensional electron gas (2DEG) with antidot lattices can be driven into the TI phase. Recently, new methods for creating topological insulating phase have been proposed^20,1^ which utilize periodic modulations and spin-orbit coupling in conventional semiconductors, including two-dimensional hole gases^20^ and conduction electrons in double quantum wells^1^. The former exploits the strong intrinsic spin-orbit coupling in the valence band to produce a TI operating at liquid nitrogen temperatures, while the latter typically operates at sub-liquid-helium temperatures due to small minigap.”

In the same section,

“We first present a general analysis for generating an artificial gauge field in a semiconductor 2DEG, then we demonstrate band inversion between neighboring subbands because of inter-subbands spin-orbit interaction (ISOI) utilizing antidot lattices created by well-developed semiconductor etching technique, and generate the TI phase with many pairs of helical edge states.”

should read:

“We first present a general analysis for generating an artificial gauge field in a semiconductor 2DEG, then we demonstrate band inversion between neighboring subbands because of inter-subbands spin-orbit interaction (ISOI)^1,2^ utilizing antidot lattices created by well-developed semiconductor etching technique, and generate the TI phase with many pairs of helical edge states.”

In the Results section,

“The third term 
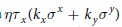
 describes the inter-subbands SOI (ISOI) obtained from the eight-band Kane model using the Löwdin perturbation theory^41^ (see Methods).”

should read:

“The third term 
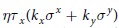
 describes the inter-subbands SOI (ISOI), which was proposed before^2^ and can be obtained from the eight-band Kane model using the Löwdin perturbation theory^41^ (see Methods)”.

In the same section,

“The SOIs in 2DEGs usually come from the asymmetry of the QWs, i.e., Rashba SOI.”

should read:

“The ISOI has been utilized in a previous proposal^1^ to generate TIs operating at sub-liquid-helium temperatures. Here we would like to emphasize that the second term in the coupling strength due to spatial variation of the Kane matrix element P(z) is absent in the previous studies^1,2^. This term can contribute considerably to the ISOI due to the spatial variation of the Kane matrix element P(z). Moreover, the even stronger SOI among higher excited subbands allows us to generate TIs operating at liquid nitrogen temperatures. The SOIs in 2DEGs usually come from the asymmetry of the QWs, i.e., Rashba SOI”.

## References

[b1] Erlingsson J. Carlos Egue. All-electron topological insulator in InAs double wells. Phys. Rev. B 91, 035312 (2015).

[b2] Bernardes J. Schliemann M. Lee J. Carlos Egues D. Los. Spin-Orbit Interaction in Symmetric Wells with Two Subbands. Phys. Rev. Lett. 99, 076603 (2007). 10.1103/PhysRevLett.99.076603 17930912

