# Supplementary material for: Artificial Gauge Field and Topological Phase in a Conventional Two-dimensional Electron Gas with Antidot Lattices
Source: Sci Rep. 2015 Oct 16;5:15266. doi: 10.1038/srep15266 (PMC4607943; doi:10.1038/srep15266)
Supplement: Supplementary Information [file srep15266-s1.pdf]

Artificial Gauge Field and Topological Phase in a Conventional Two-dimensional Electron Gas with Antidot Lattices

Likun Shi[1], Wenkai Lou[1], F. Cheng[1], Y. L. Zou[1], Wen Yang[2] and Kai Chang[1]
[1]*SKLSM, Institute of Semiconductors, Chinese Academy of Sciences, P.O. Box 912, Beijing 100083, China and*
[2]*Beijing Computational Science Research Center, Beijing 100094, China*

Supplementary Note 1: Effective gauge field in spin-orbit coupled systems

In the spin-orbit coupled system, adopting the Born-Oppenheimer approximation, the total Hamiltonian can be divided into two parts:

$$\hat{H}(\hat{\mathbf{k}},\hat{\mathbf{r}},\hat{\boldsymbol{\sigma}}) = \hat{H}_{\rm orb}(\hat{\mathbf{k}},\hat{\mathbf{r}}) + H_{\rm s-o}(\hat{\mathbf{k}},\hat{\boldsymbol{\sigma}}),$$

where $\hat{H}_{\rm orb}(\hat{\mathbf{k}},\hat{\mathbf{r}})$ stands for the intra-band (slow) orbital motion part, and $H_{\rm s-o}(\hat{\mathbf{k}},\hat{\boldsymbol{\sigma}})$ is the inter-band (fast) spin-orbit part. For a given eigenvalue $\mathbf{k}$ of the momentum operator $\hat{\mathbf{k}}$, the eigenstates of the spin-orbit part is denoted by $|n(\mathbf{k})\rangle$ ($n = 1, 2, \cdots$) and the corresponding eigenenergies are $\epsilon_n(\mathbf{k})$. We work in the momentum representation of the orbital part and expand the eigenstate of $\hat{H}(\hat{\mathbf{k}},\hat{\mathbf{r}},\hat{\boldsymbol{\sigma}})$ in this representation, $|\Psi(\mathbf{k})\rangle \equiv \langle\mathbf{k}|\Psi\rangle$, as

$$|\Psi(\mathbf{k})\rangle = \sum_{n,\mathbf{k}} \phi_n(\mathbf{k})|\chi_n(\mathbf{k})\rangle.$$

In the momentum representation, we have $\hat{\mathbf{r}} = i\nabla_{\mathbf{k}}$ and $\hat{\mathbf{k}} = \mathbf{k}$. Substituting into $\hat{H}(\mathbf{k},\hat{\mathbf{r}},\hat{\boldsymbol{\sigma}})|\Psi(\mathbf{k})\rangle = E|\Psi(\mathbf{k})\rangle$, we have

$$\sum_n H_{m,n}(\mathbf{k})\phi_n(\mathbf{k}) = E\phi_m(\mathbf{k}),$$

where

$$\begin{aligned} H_{m,n}(\mathbf{k}) &\equiv \delta_{m,n}\epsilon_m(\mathbf{k}) + \langle\chi_m(\mathbf{k})|\hat{H}_{\rm orb}(\mathbf{k},\hat{\mathbf{r}})|\chi_n(\mathbf{k})\rangle \\ &= \delta_{m,n}\epsilon_m(\mathbf{k}) + \hat{H}_{\rm orb}(\mathbf{k},\hat{\mathbf{r}} - \mathbf{A}_{m,n}(\mathbf{k})) \end{aligned}$$

contains a pure gauge $\mathbf{A}_{m,n}(\mathbf{k}) \equiv -i\langle\chi_m(\mathbf{k})|\nabla_{\mathbf{k}}|\chi_n(\mathbf{k})\rangle$. By now the above equation is still exact. Now we make the Born-Oppenheimer approximation and consider adiabatic transport, i.e., neglect the off-diagonal coupling between different spin-orbit energy bands, to arrive at the single-band description

$$H_n(\mathbf{k},\hat{\mathbf{r}}'_n)\phi_n(\mathbf{k}) = E\phi_n(\mathbf{k}),$$

where the effective single-band Hamiltonian on the orbital motion

$$H_n(\mathbf{k},\hat{\mathbf{r}}'_n) = \epsilon_n(\mathbf{k}) + \hat{H}_{\rm orb}(\mathbf{k},\hat{\mathbf{r}}'_n),$$

where $\hat{\mathbf{r}}'_n = \hat{\mathbf{r}}_n - \mathbf{A}_n(\mathbf{k})$ contains an effective gauge field for the slow orbital motion:

$$\mathbf{A}_n(\mathbf{k}) = -i\left\langle\chi_n(\mathbf{k})\right|\nabla_{\mathbf{k}}\left|\chi_n(\mathbf{k})\right\rangle.$$

Specifically, for the BHZ model of 2D TIs in the presence of an in-plane uniform electric field, the slow orbital part is

$$\hat{H}_{\rm orb}(\hat{\mathbf{k}},\hat{\mathbf{r}}) = C - Dk^2 - e\mathbf{E}\cdot\mathbf{r},$$

and the fast spin-orbital part is

$$H_{\rm s-o}(\hat{\mathbf{k}},\hat{\boldsymbol{\sigma}}) = \begin{pmatrix} M - Bk^2 & Ak_+ & 0 & 0 \\ Ak_- & -M + Bk^2 & 0 & 0 \\ 0 & 0 & M - Bk^2 & Ak_- \\ 0 & 0 & Ak_+ & -M + Bk^2 \end{pmatrix},$$

and

$$A_{x,n} = s_n \frac{k_y}{2k^2}\left[1 + t_n \frac{Bk^2 - M}{[A^2k^2 + (M - Bk^2)^2]^{1/2}}\right],$$

$$A_{y,n} = s_n \frac{k_x}{2k^2}\left[1 + t_n \frac{Bk^2 - M}{[A^2k^2 + (M - Bk^2)^2]^{1/2}}\right].$$

where $s_n = \pm 1$ for spin-up/down block, and $t_n = \pm 1$ for the electron in conduction/valence band ($n = 1, 2, 3, 4$). The effective vector potential leads to the non-trivial effective gauge field with the strength

$$\begin{aligned} F_{xy,n}(\mathbf{k}) &\equiv i[x', y'] = i[i\partial_{k_x} - A_x, i\partial_{k_y} - A_y] \\ &= (\nabla_{\mathbf{k}} \times \mathbf{A})_z = \lambda_n \frac{A^2(M + Bk^2)}{2[A^2k^2 + (M - Bk^2)^2]^{3/2}}, \end{aligned}$$

where $\lambda_n = s_n \times t_n = \pm 1$ ($n = 1, 2, 3, 4$). Within the Born-Oppenheimer approximation, the equation of motion for the n-th band can be written as

$$\begin{aligned} \dot{x}'_n &= \frac{\partial H_n}{\hbar \partial k_x} + F_{xy,n}(k)\dot{k}_y, \\ \dot{y}'_n &= \frac{\partial H_n}{\hbar \partial k_x} - F_{xy,n}(k)\dot{k}_x, \\ \dot{k}_i &= eE_i/\hbar, \end{aligned}$$

we can see that the gauge field strength $F_{xy,n} = (\nabla_{\mathbf{k}} \times \mathbf{A})_z$ acts as a Lorentz force in the k-space, acting on spin-up and spin-down electrons in opposite directions, which is perpendicular to electron momentum.

Supplementary Note 2: Effective Hamiltonian reduced numerically from the eight-band Kane model

At the Γ point, the wave functions in the eight-band $\boldsymbol{k} \cdot \boldsymbol{p}$ Hamiltonian are

$$\psi_1 = \begin{pmatrix} F_1^{(1)}(z) \\ F_2^{(1)}(z) \\ F_3^{(1)}(z) \\ F_4^{(1)}(z) \\ \vdots \\ F_8^{(1)}(z) \end{pmatrix} e^{ik_{//}\cdot\vec{r}}, \psi_2 = \begin{pmatrix} F_1^{(2)}(z) \\ F_2^{(2)}(z) \\ F_3^{(2)}(z) \\ F_4^{(2)}(z) \\ \vdots \\ F_8^{(2)}(z) \end{pmatrix} e^{ik_{//}\cdot\vec{r}}, \cdots\cdots, \psi_m = \begin{pmatrix} F_1^{(m)}(z) \\ F_2^{(m)}(z) \\ F_3^{(m)}(z) \\ F_4^{(m)}(z) \\ \vdots \\ F_8^{(m)}(z) \end{pmatrix} e^{ik_{//}\cdot\vec{r}},$$

which can be obtained by solving the secular equation $H^{(0)}_{8\times 8}\psi_m = E_m\psi_m$.

Considering the two lowest electron subbands, we obtain the effective two-dimensional Hamiltonian by averaging the z component in the Hamiltonian

$$H_{\text{eff}}(\mathbf{k}_{\|}) = \langle \Psi(z)|H|\Psi(z)\rangle,$$

where the matrix element of the Hamiltonian is

$$\begin{aligned} \langle H_{\text{eff}}\rangle_{mn} &= \left\langle \psi^{(m)} \right| H_{8\times 8} \left| \psi^{(n)} \right\rangle \\ &= \int \begin{pmatrix} F_1^{*(m)}(z) & F_2^{*(m)}(z) & \cdots\cdots & F_7^{*(m)}(z) & F_8^{*(m)}(z) \end{pmatrix} (H)_{8\times 8} \begin{pmatrix} F_1^{(n)}(z) \\ F_2^{(n)}(z) \\ \vdots \\ F_7^{(n)}(z) \\ F_8^{(n)}(z) \end{pmatrix} dz \\ &= \sum_{i,j=1}^{8} \langle F_i^{(m)}(z)|H_{ij}|F_j^{(n)}(z)\rangle. \end{aligned}$$

The Hamiltonian can be divided into

$$H = H^{(0)} + H',$$
$$H'\left(\mathbf{q}, \hat{k}_z\right) = \alpha(\mathbf{q}) + \beta(\mathbf{q})\hat{k}_z + \gamma(\mathbf{q})\hat{k}_z^2.$$

Then we have

$$\langle H_{\text{eff}}\rangle_{mn} = E_m\delta_{m,n} + \sum_{i,j=1}^{8}\left\langle F_i^{(m)}(z)\right|[\alpha(\mathbf{q})]_{ij}|F_j^{(n)}(z)\rangle + \langle F_i^{(m)}(z)|[\beta(\mathbf{q})]_{ij}\hat{k}_z|F_j^{(n)}(z)\rangle$$
$$+\langle F_i^{(n)}(z)|[\gamma(\mathbf{q})]_{ij}\hat{k}_z^2|F_j^{(n)}(z)\rangle.$$

The contribution of the subbands other than the two lowest electron subbands should also be considered in the reducing process, which can be done by using Löwdin perturbation theory. We include the lowest 20 electron subbands and 54 highest hole subbands in the QW respectively and divide them into the weakly coupled subsets S_1 and S_2. The set S_1 includes the two lowest electron subbands $|\chi_1\rangle$ and $|\chi_2\rangle$, the other subbands are included in the set S_2. The Hamiltonian is reduced into set S_1 using the Löwdin perturbation method,

$$H^{(2)}_{mm'} = \frac{1}{2}\sum_l H'_{ml}H'_{lm'}\left[\frac{1}{E_m - E_l} + \frac{1}{E_{m'} - E_l}\right],$$

where the indices m correspond to states in the set A, the indices l correspond to states in the set B, and

$$H'_{ml} = \langle\psi_m|H'|\psi_l\rangle.$$

Finally we obtain the effective two-dimensional Hamiltonian in the basis $|\chi_1,\uparrow\rangle$, $|\chi_2,\downarrow\rangle$, $|\chi_1,\downarrow\rangle$, $|\chi_2,\uparrow\rangle$:

$$H_{\text{eff}}^{4\times4} = \begin{pmatrix} E_1 + B_1\cdot k_\parallel^2 & Ak_+ & 0 & 0 \\ Ak_- & E_2 + B_2\cdot k_\parallel^2 & 0 & 0 \\ 0 & 0 & E_1 + B_1\cdot k_\parallel^2 & Ak_- \\ 0 & 0 & Ak_+ & E_2 + B_2\cdot k_\parallel^2 \end{pmatrix},$$

where

$$E_1 = 0.05351\text{eV},$$
$$B_1 = 0.86571\text{eV}\cdot\text{nm}^2,$$
$$E_2 = 0.14396\text{eV},$$
$$B_2 = 0.67969\text{eV}\cdot\text{nm}^2,$$
$$A = 0.01041\text{eV}\cdot\text{nm}.$$

In order to examine the validity of the 4-band Hamiltonian, we plot the band structure of the GaAs/$\text{In}_x\text{Ga}_{1-x}$As/GaAs parabolically graded quantum well calculated by the 4-band model and compare it with the eight-band $\boldsymbol{k}\cdot\boldsymbol{p}$ model (see Fig. 2a in the manuscript). One can see clearly that the band structure obtained from the 4-band model [the solid lines in Fig. 2a] is in good agreement with that obtained from the eight-band model [the dashed lines in Fig. 2a].
